# P-1655. Impact of an Educational Leaflet on Urinary Tract Infections, Asymptomatic Bacteriuria, and Antibiotics for Adults ≥65 years

**DOI:** 10.1093/ofid/ofae631.1821

**Published:** 2025-01-29

**Authors:** Alistair Thorpe, Rachael A Lee, Julia E Szymczak, Madeline C Farrell, Karen Fong, Brandi M Muller, Andrea T White, Angela Fagerlin, Valerie Vaughn

**Affiliations:** Spencer Fox Eccles School of Medicine at University of Utah, Salt Lake City, Utah; University of Alabama at Birmingham, Birmingham, AL; University of Utah School of Medicine, Salt Lake City, Utah; No current affiliation, Westbrook, Connecticut; University of Utah Health, Salt Lake City, Utah; University of Utah, Haddon Township, New Jersey; University of Utah, Haddon Township, New Jersey; Spencer Fox Eccles School of Medicine at University of Utah, Salt Lake City, Utah; University of Utah Medical School, Salt Lake City, UT

## Abstract

**Background:**

Adults ≥65yrs are at high risk of harm from antibiotic overuse due to misdiagnosis of asymptomatic bacteriuria (ASB) as urinary tract infection (UTI). Alongside strategies to improve clinician prescribing, patients should be educated on ASB and empowered to discuss harms and benefits of antibiotic treatment. Previously, we used a user-centered design process to develop a patient-focused educational leaflet on UTI, ASB, and antibiotic use (Figure 1). Here, we tested whether the leaflet improved patients’ knowledge about ASB and willingness to avoid unnecessary antibiotics.

**Figure 1.**
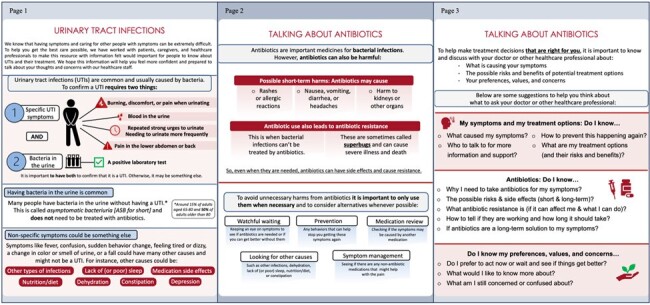
A three-page antibiotic education designed with patients, caregivers, and clinicians.

**Methods:**

In an online survey experiment of US adults ≥65yrs, respondents read a scenario of themselves as an asymptomatic patient with a positive urine test during prescreening for non-urologic surgery. Respondents were randomized to one of four experimental conditions which varied educational leaflet provision and the surgeons’ treatment recommendation (Figure 2). Outcome measures included whether respondents thought they (as the patient described in the case) had a UTI, how comfortable they would be not taking antibiotics, and their knowledge about UTIs, ASB, and antibiotics

**Figure:**
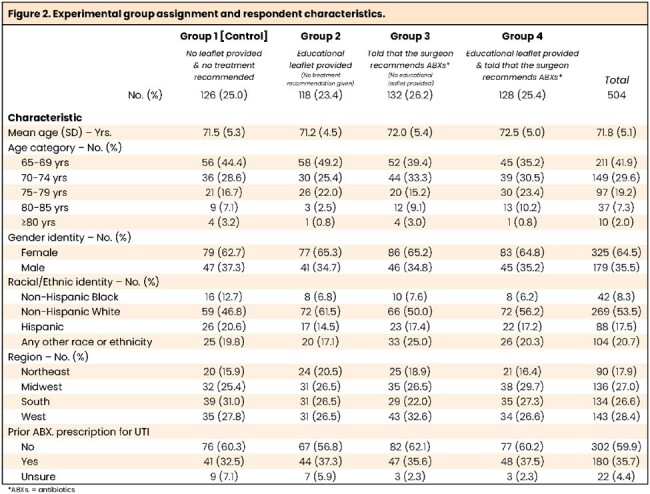

**Results:**

Figure 2 shows characteristics of the 504 study respondents [completion=89%].

Compared to those not provided the educational leaflet, respondents shown the educational leaflet were less likely to believe they had UTI (*p*< .001), were more comfortable not taking antibiotics (*p*< .001), and answered more knowledge questions correctly (*p*< .001) (Figures 3&4).

Conversely, respondents who were told the surgeon recommends antibiotics were more likely to believe they had UTI (*p*< .001) and less comfortable with not taking antibiotics (*p*=.013) but did not differ in their knowledge (*p*=.096).

**Figure:**
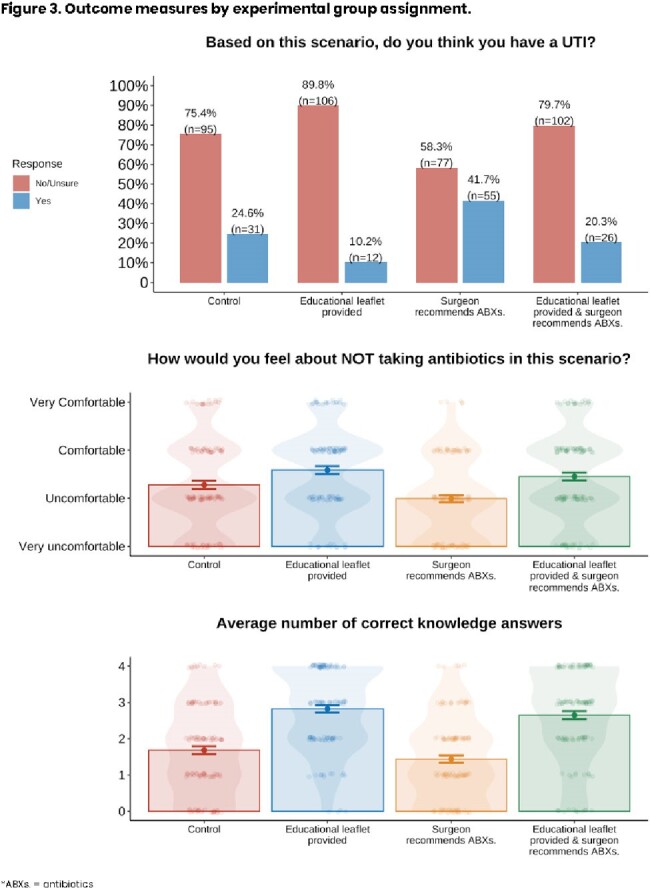

**Conclusion:**

A patient-centered educational leaflet, designed with patients, caregivers, and clinicians, was effective in aligning antibiotic treatment preferences with clinical guidelines and improving knowledge about UTI, ASB, and antibiotics. Findings offer important preliminary evidence on the potential for patient/caregiver-focused education to help prepare patients/caregivers to engage in treatment decisions and contribute to reducing antibiotic overuse and its associated harms.

**Figure:**
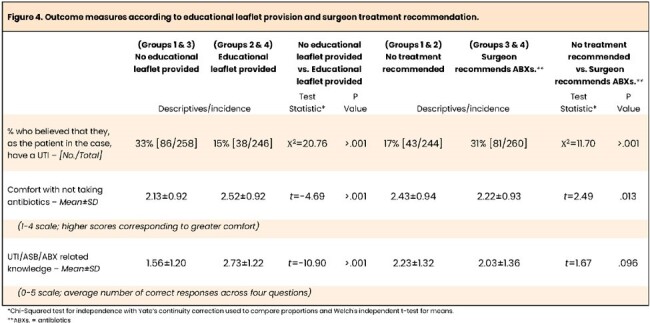

**Disclosures:**

**All Authors**: No reported disclosures

